# Improved Metagenomic Taxonomic Profiling Using a Curated Core Gene-Based Bacterial Database Reveals Unrecognized Species in the Genus *Streptococcus*

**DOI:** 10.3390/pathogens9030204

**Published:** 2020-03-10

**Authors:** Mauricio Chalita, Sung-min Ha, Yeong Ouk Kim, Hyun-Seok Oh, Seok-Hwan Yoon, Jongsik Chun

**Affiliations:** 1 Interdisciplinary Program in Bioinformatics, Seoul National University, Seoul 08826, Korea; mauricio@snu.ac.kr (M.C.); kim331@snu.ac.kr (Y.O.K.); araohs@snu.ac.kr (H.-S.O.); 2 ChunLab Inc., Seoul 06725, Korea; smha118@snu.ac.kr (S.-m.H.); seokhwan.yoon@chunlab.com (S.-H.Y.)

**Keywords:** shotgun metagenomics, taxonomy prediction, *Streptococcus*, genomospecies

## Abstract

Shotgun metagenomics is of great importance in order to understand the composition of the microbial community associated with a sample and the potential impact it may exert on its host. For clinical metagenomics, one of the initial challenges is the accurate identification of a pathogen of interest and ability to single out that pathogen within a complex community of microorganisms. However, in absence of an accurate identification of those microorganisms, any kind of conclusion or diagnosis based on misidentification may lead to erroneous conclusions, especially when comparing distinct groups of individuals. When comparing a shotgun metagenomic sample against a reference genome sequence database, the classification itself is dependent on the contents of the database. Focusing on the genus *Streptococcus*, we built four synthetic metagenomic samples and demonstrated that shotgun taxonomic profiling using the bacterial core genes as the reference database performed better in both taxonomic profiling and relative abundance prediction than that based on the marker gene reference database included in MetaPhlAn2. Additionally, by classifying sputum samples of patients suffering from chronic obstructive pulmonary disease, we showed that adding genomes of genomospecies to a reference database offers higher taxonomic resolution for taxonomic profiling. Finally, we show how our genomospecies database is able to identify correctly a clinical stool sample from a patient with a streptococcal infection, proving that genomospecies provide better taxonomic coverage for metagenomic analyses.

## 1. Introduction

Taxonomy classification and quantification of each bacterial species within a shotgun metagenomic sample is a primary goal of most microbiome analyses, which still can be a complicated task. Sequencing of a given metagenomic sample generates a large number of sequence reads that are then fed to a bioinformatic process involving searches of each read against the reference sequence database. The general, albeit important, assumption is that reference-based databases currently available for shotgun metagenomics contain the reference genome sequences that we are interested in. When those references are absent, most classifiers (software that can be used to taxonomically profile a shotgun metagenomic sample), based on sequence identity, will match the reads from the metagenomic sample to the closest reference available in the database. The user, not knowing precisely which bacterial species are present in the sample, may assume that the taxonomic identification is accurate. Adding more references to a database can be a complex task as well; adding new genome sequences to a reference database not only will increase the database size, but also will increase the number of comparisons that the classifier has to perform between the references and each read within the sample. Alternatives to the use of full genomic sequences, MetaPhlAn2 [1] proposes the use of marker genes, which are gene sequences that only occur once within a specific taxon, in this case at the species level. However, this approach assumes that we have a reference genome of every species within a genus, if a new species is sequenced, all marker genes must be recalculated. To solve this limitation, we propose the use of UBCG (up-to-date bacterial core gene) sequences [2] as reference for shotgun metagenomics. UBCG sequences are core genes that are defined as single-copy and homologous sequences; they are present in almost all bacterial species. At present, a total of 92 core genes are used for the version 3.0 of the UBCG pipeline, and regardless of the assembly level of a genome or its completeness, we can assume that a reference is complete if all 92 core genes are present in the genome. 

To demonstrate the usefulness of these 92 core gene sequences in the use of shotgun metagenomic profiling, we focus on the genus *Streptococcus*, a highly diverse taxon in the phylum *Firmicutes*. The genus comprises a large number of species with a variety of pathogenic potentials to humans and animals including opportunistic pathogens like *Streptococcus oralis* [3], and harmless or even considered probiotic *Streptococcus thermophilus* [4]. *Streptococcus*-caused sepsis is often associated with *Streptococcus pneumoniae* and *Streptococcus pyogenes* [5]. *Streptococcus mitis* has been associated to several clinical diseases like viridians group streptococci (VGS) shock syndrome in cancer patients [6]. *Streptococcus pneumoniae* is commonly found on sputum samples of patients with community-acquired pneumonia, so rapid detection in a clinical setting is of high importance [7].

As of November 2019, a total of 88 validly named species and 114 genomospecies of *Streptococcus* are recorded in the EzBioCloud reference database [8]; a genomospecies is defined as a tentatively novel species that is supported by genomic evidence, such as average nucleotide identity (ANI) [9,10,11]. Most publicly available databases for shotgun metagenomics do not encompass these genomospecies references since they are not formally described and named.

Here, by extracting all 92 core genes for each species available in the EzBioCloud database, including 114 genomospecies of *Streptococcus*, we built two core gene-based databases, one containing only validly named species (KrakenUBCG-VNS) and a second one containing both validly named species and genomospecies (KrakenUBCG). By focusing on the genus *Streptococcus*, using synthetic and real clinical datasets, we demonstrate how bacterial core genes can be a better alternative as reference sequence databases for metagenomic taxonomic profiling, and finally we show the impact that a metagenomic taxonomic profile will have when including or excluding genomospecies from two core gene-based databases.

## 2. Results and Discussion

### 2.1. Building a Core Gene-Based Reference Database

**Figure 1:**
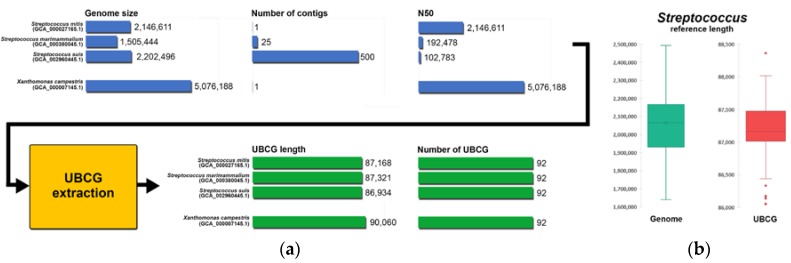
(**a**) Extracting process of UBCG (up-to-date bacterial core gene) sequences from genomic sequences with variable length, number of contigs, and N50 values; (**b**) range and the median of the reference size for all the genomes and core genes for the species and genomospecies contained in the genus *Streptococcus*.

Bacterial genomic sequences on public databases have a diverse range of genomic statistics, such as reference size, number of contigs, assembly status (complete, chromosome, scaffold, contig), N50 values among others. For some species, a high-quality assembly may not be available. When using these references for metagenomic shotgun profiling, this variation may provide a bias for higher quality and complete genomes, making abundance quantification unreliable, particularly for genomes that may be considered incomplete. To avoid this, we extracted UBCG sequences [2] (92 core genes) from full genomic references from the EzBioCloud database [8]. Regardless of the assembly status or genome length, extracting these core genes will remove any bias based on genome quality. Figure 1a shows three streptococcal references with variable genome size, number of contigs, and N50 value. When extracting the UBCG sequences, all the references end up being represented by the same number of genes (92) and their sequence size is near identical. This shows that regardless of having a complete genome with one contig, or a contig assembly with 500 contigs, UBCG will provide an unbiased representation for any bacterial reference regardless of their assembly status, making detection and abundance estimation more reliable. Figure 1b shows how genomic sequences from a variable length size for the genus *streptococcus* can be translated into 92 UBCG sequences with a narrower difference in sequence length. After extracting all 92 UBCG sequences from the bacterial references, we built two Kraken [12] based pipelines containing these sequences as references, one with just validly named bacterial species (KrakenUBCG-VNS) and another one containing also genomospecies references (KrakenUBCG). Table 1 shows the difference between these two pipelines, highlighting their core algorithm and the number of bacterial and streptococcal species that their databases represent.

### 2.2. Evaluation of Pipelines Using Synthetic Metagenomes

**Figure 2:**
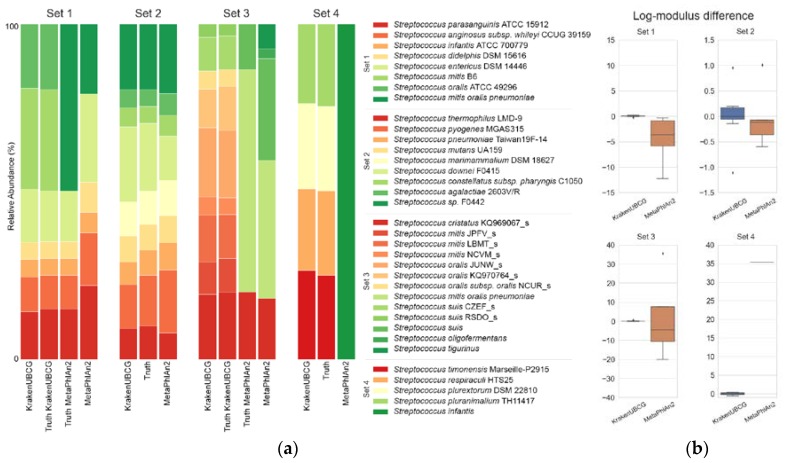
(**a**) Taxonomic prediction made by KrakenUBCG and MetaPhlAn2 for 4 synthetic metagenome sets containing species from *Streptococcus*; (**b**) Log-modulus difference between the predicted abundance and the expected abundance (truth).

To compare the classification accuracies by bacterial core genes as reference sequences, we created four different synthetic metagenomic datasets containing only species from the genus *Streptococcus* using the InSilicoSeq pipeline [13] and proceeded to compare the results with MetaPhlAn2. Supplementary Table S2 contains the description and accession number of the genomes used on these datasets along with their true abundances. Figure 2a shows the predicted abundances for all four datasets using the KrakenUBCG pipeline and MetaPhlAn2, respectively, along with the true abundance of the synthetic sets. In order to compare some of MetaPhlAn2’s predictions to our pipeline KrakenUBCG, we included an alternative truth for some sets, since MetaPhlAn2 clusters several *Streptococcus* species into a single taxon group. Figure 2b shows the log-modulus difference of abundance prediction made by MetaPhlAn2 and the KrakenUBCG pipeline. The first synthetic set consists of seven different species of public assembled genomes from the NCBI database, which were included in both pipelines. MetaPhlAn2’s prediction was compared against a grouped truth, where all the genomes that belong to *S. mitis* and *S. oralis* are clustered into a single group (*S. mitis*, *S. oralis*, and *S. pneumoniae;* referred as mitis group hereafter). Both MetaPhlAn2 and KrakenUBCG were able to identify correctly the presence of all species of *Streptococcus* contained in the sample, however MetaPhlAn2’s marker gene database cannot distinguish between the species that are contained in the mitis group. Relative abundance predictions were less consistent for MetaPhlAn2, as it seems to be unable to predict correct abundances for the mitis group. While the expected abundance for *S. mitis* and *S. oralis* was expected at 30% and 20% respectively, MetaPhlAn2 only assigned a total abundance of 21.2% (mitis group), much lower than the expected 50%. KrakenUBCG, on the other hand, accurately predicted 29.9% and 19.3% relative abundance, respectively. Lower abundance prediction by MetaPhlAn2 for the mitis group indicates its overprediction of abundance for the remaining species, most notably *Streptococcus entericus* with a predicted abundance of 26.3% instead of an expected abundance of 15%, while KrakenUBCG accurately predicted its abundance at 15.8%.

Synthetic dataset 2 consists of nine species of *Streptococcus* that were contained in both databases while the genome assemblies representing each species were different. Both pipelines predicted correctly the presence of all the species contained in the sample, and while the KrakenUBCG pipeline contains different reference assemblies for all nine species, it was able to detect the presence of all nine species correctly. KrakenUBCG’s abundance predictions were more accurate than MetaPhlAn2’s, for the 20% expected abundance for *Streptococcus downei,* KrakenUBCG and MetaPhlAn2 predicted 22.2% and 13.19%, respectively, making KrakenUBCG overpredicting the abundance by 2.2% while MetaPhlAn2 underpredicted the abundance by 6.81%. *Streptococcus pyogenes* had an expected abundance of 15% with a predicted abundance of 13% and 18.64% for KrakenUBCG and MetaPhlAn2, respectively. For the remaining species, KrakenUBCG’s worst prediction was for *Streptococcus pneumoniae*, with a prediction of 6.72% against an expected abundance of 7.5%, while MetaPhlAn2’s worst prediction was *Streptococcus agalactiae*, with a predicted abundance of 6.5% against an expected abundance of 5%. Overall, both pipelines performed better than expected, however KrakenUBCG performed favorably over MetaPhlAn’s abundance prediction even though it did not contain the assemblies contained in the metagenomic sample.

The third set contains the assemblies of nine different genomes from the genus *Streptococcus*. Those genomes are annotated as genomospecies in the KrakenUBCG pipeline. MetaPhlAn2 contains the same assemblies, however, some of them are annotated as the same species under the NCBI database, so we expect MetaPhlAn2’s prediction to follow that annotation. Those annotations are as follows: genomospecies KQ969067_s is annotated at the NCBI database as *Streptococcus cristatus*; JPFV_s, LBMT_s, and NCVM_s as *Streptococcus mitis*; JUNW_s, KQ970764_s, and NCUR_s as *Streptococcus oralis*; and finally, CZEF_s and RSDO_s as *Streptococcus suis*. MetaPhlAn2 is also unable to distinguish between *Streptococcus oralis* and *Streptococcus mitis*, so while the KrakenUBCG prediction is expected to predict nine different genomospecies (KQ969067_s, JPFV_s, LBMT_s, NCVM_s, JUNW_s, KQ970764_s, NCUR_s, CZEF_s, and RSDO_s), MetaPhlAn2 is only expected to predict three distinct species (*Streptococcus suis*, *Streptococcus cristatus*, and mitis group), and while it predicted the presence of those three taxa correctly, it also predicted the presence of two other *Streptococcus* species (false positives). KrakenUBCG’s prediction, however, predicted correctly the presence of all nine genomospecies present in the sample. Abundance predictions for MetaPhlAn2 particularly for the mitis group were low, with an abundance prediction of 40.93% against an expected abundance of 66%. For *Streptococcus suis*, MetaPhlAn2 predicted 30.21% against an expected 14% abundance. KrakenUBCG’s worst abundance prediction was for the genomospecies KQ970764_s with 11.5% against an expected abundance of 13%.

Finally, set 4 consists of species present on the KrakenUBCG pipeline but absent on the MetaPhlAn2 database. MetaPhlAn2 predicted a single species (*Streptococcus infantis*) which is not present in the sample, and while it was expected that MetaPhlAn2 would not be able to predict correctly any of the species due to their absence in their database, it was not unable to predict the presence of more than one species. KrakenUBCG predicted all four species present, as for abundance predictions, each species had an expected abundance of 25% each, and KrakenUBCG predicted 26.4%, 24.2%, 25.3%, 23.9% abundance for *Streptococcus timonensis*, *Streptococcus respiraculi*, *Streptococcus plurextorum*, and *Streptococcus pluranimalium*, respectively.

Using four synthetic metagenomic samples containing full genome reads from *Streptococcus*, we demonstrated that using core genes as references not only predict accurate species composition, but also showed little difference when comparing predicted relative abundance versus the absolute abundance. We also showed that MetaPhlAn2 suffers from lower accuracy in predicting relative abundance, probably because its marker gene references are uneven in coverage, especially for the mitis group.

### 2.3. Chronic Obstructive Pulmonary Disease Samples

Chronic obstructive pulmonary disease (COPD) is an obstructive lung disease where the affected individual suffers from long-term breathing problems and airflow. Cameron et al. studied the association between the microbiome and COPD by comparing the metagenomic profiles of two different groups, patients with COPD and ‘healthy’ smoking controls [14], and showed significant changes in abundance of bacterial species, particularly in the genus *Streptococcus*. They also found the presence of *Streptococcus pneumoniae* in all the samples. Using KrakenUBCG with 201 different *Streptococcus* reference genomes and MetaPhlAn2’s database, we profiled all the samples and compared the prediction of *Streptococcus* at genus and species level.

**Figure 3:**
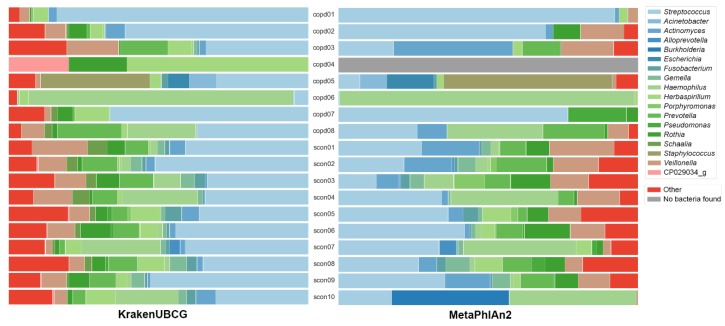
Predictions of taxonomic abundance at genus level between the KrakenUBCG database with genomospecies and the MetaPhlAn2 database.

Figure 3 shows the abundance prediction at genus level by both KrakenUBCG and MetaPhlAn2. While the presence of other genera is variable among the databases, they are outside the scope of this study and will not be discussed in detail, however, it is important to mention that the number of classified reads for other genera will also have a direct impact on the predicted abundance of *Streptococcus*. As an example, this can be observed on sample copd04, MetaPhlAn2 was unable to detect the presence of any bacteria, while KrakenUBCG detected the presence of three distinct genera.

**Figure 4:**
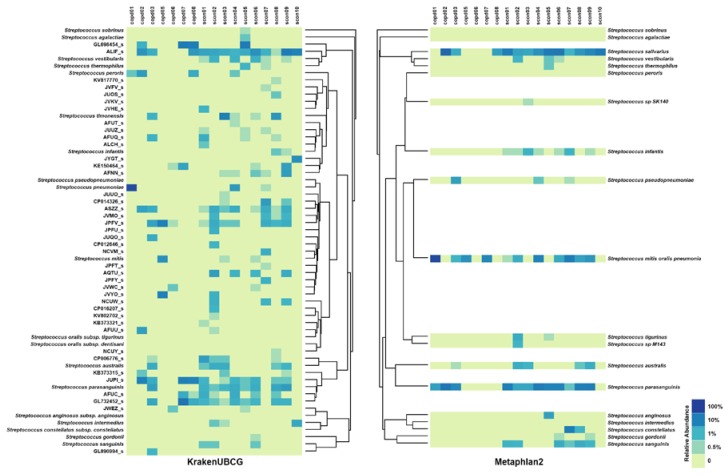
Species predicted for *Streptococcus* using the KrakenUBCG and the MetaPhlAn2 pipeline demonstrates untapped *Streptococcus* diversity revealed by KrakenUBCG remain obscured by MetaPhlAn2. An ANI dendrogram can be seen in the middle of the heatmaps. References from MetaPhlAn2 were matched against the references from KrakenUBCG and aligned accordingly.

Figure 4 shows heatmaps for predictions made by KrakenUBCG and MetaPhlAn2 along with their respective ANI dendrogram. Only samples that contained any *Streptococcus* in either of the predictions were included. References from the MetaPhlAn2 database were matched with the KrakenUBCG pipeline and aligned on the heatmap accordingly. Predictions with a minimum of 0.5% abundance are shown, also only the species and genomospecies that were contained in at least one of the samples were included. 

One of the major differences between both pipelines is the mitis group on MetaPhlAn2, which is covered by 21 different species and genomospecies at KrakenUBCG, showing clear differences as expected. On KrakenUBCG, only 3 samples showed the presence of *S. pneumoniae*, and another 3 showed presence of *S. mitis*, but none of the samples showed the presence of both at the same time; genomospecies CP016207_s, KV802702_s, KB373321_s, JPFY_s, JPFT_s, NCVM_s, CP012646_s, JUQO_s, JPFU_s, and JUUO_s were predicted only once among the samples, while the remaining genomospecies were found in two or more of the samples. While MetaPhlAn2 found zero presence of bacteria in the mitis group for sample copd02, copd06, scon05, and scon10, KrakenUBCG found the presence of ASZZ_s and AFUU_s for copd02, and JPFV_s and JVWC_s for copd06. For samples scon05 and scon10, KrakenUBCG did not find the presence of any species or genomospecies closer to *S. pneumoniae* or *S. mitis* matching MetaPhlAn2’s prediction. Looking at some samples individually we can observe distinct predictions, for example, MetaPhlAn2 predicted only one species of *Streptococcus* (*S. salivarius*) with a 17.5% abundance, while KrakenUBCG found three distinct species (ALIF_s, JYGT_s and *S. intermedius*) with a total abundance of 20.7%. If we analyze each sample individually, we can observe more differences than coincidences. These differences are the result of the lack of references on MetaPhlAn2’s database, and the argument can be made that only formally named species should be added to a reference database. However, if MetaPhlAn2’s authors decide to add genomospecies references to their database, they would require to recalculate all gene markers to those genus affected with newly added genomospecies, and even if somehow, these recalculations can be done within reasonable computational time, we can already observe that these gene markers cannot differentiate between three already recognized species (*S. mitis, S. oralis* and *S. pneumoniae*), so adding any more references may result in more larger groups of species, just like the mitis group.

**Figure 5:**
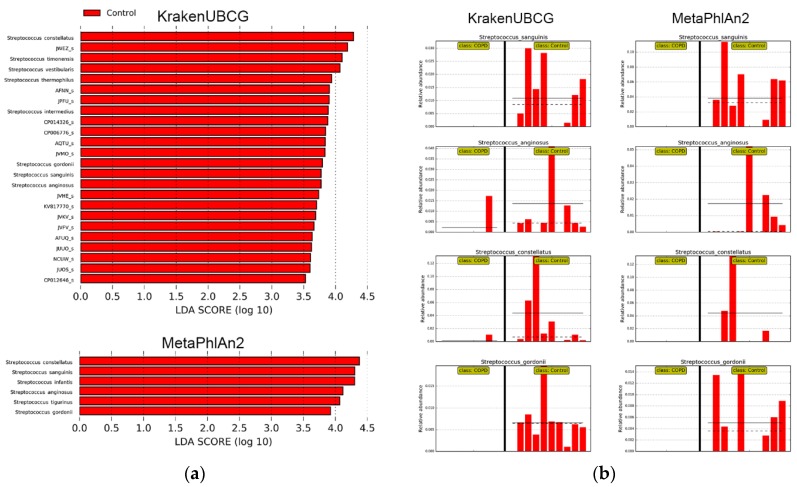
(**a**) Taxonomic biomarkers found by LEfSe using KrakenUBCG and MetaPhlAn2; (**b**) Biomarker features found in common between KrakenUBCG and MetaPhlAn2.

Figure 5a shows the taxonomic biomarkers found by LEfSe [15] by KrakenUBCG and MetaPhlAn2. While the study was not aimed at finding associations between COPD and healthy controls, our analyses demonstrated that any kind of conclusion driven by the use of distinct databases may lead to different findings. Both databases found a great diversity of streptococcal species found in the control samples that were absent in the COPD samples. KrakenUBCG found 24 streptococcal biomarkers (8 validly named species and 16 genomospecies) contained in the control metagenomic samples while MetaPhlAn2 detected 6 streptococcal biomarkers, demonstrating the impact that a high species and genomospecies coverage would have on the biomarker discovery process. Figure 5b shows four biomarkers found by both pipelines while the remaining biomarkers were exclusive to each pipeline. These common biomarker predictions done by both pipelines show that regardless of the core algorithm or database, when a species is predicted accurately, the process for taxonomic biomarker discovery can reliable. However, it also shows how the absence of streptococcal species on MetaPhlAn2’s database would miss several biomarkers that were detected by KrakenUBCG (Figure 5a). Absence of species may also incur on false taxonomic predictions, particularly when a reference species has no genomically close species. For example, MetaPhlAn2 detected *Streptococcus infantis* as a biomarker, however this may be explained by Figure 4, where it can be seen that MetaPhlAn2’s database lacks reference species genomically close to its genome, while KrakenUBCG detected the presence of *Streptococcus infantis* only in two control samples while the rest of the prediction was assigned to other genomically close genomospecies. This example showcases how an outdated database might impact metagenomic profiling and biomarker discovery, since the lack of genomic diversity around the reference of *Streptococcus infantis* will potentially incur in taxonomic false positives and result in a false biomarker prediction.

Here, by comparing classification results using samples from COPD patients with our genomospecies database KrakenUBCG, we found the presence of thirty-three different genomospecies and nineteen species across the samples. On the other hand, MetaPhlAn2 only found the presence of seventeen species of *Streptococcus*, demonstrating the importance of keeping a database up to date (Figure 4). This finding indicates that lack of all species and genomospecies in the database being used and their taxonomies inconsistencies can exert a dramatic impact on the discovery of reliable biomarkers and biodiversity estimates. While MetaPhlAn2’s algorithmic predictions may not be incorrect, their use of an outdated database with an incorrect taxonomy annotation may have an impact on their prediction performance, specifically since they rely on that taxonomy structure in order to generate their reference marker genes. Additionally, the use of marker genes as reference sequences makes the process of updating their database difficult. While generating those marker genes itself requires substantial time and efforts, the process is further complicated by the fact that every time when a new species is added, all the marker genes have to be recalculated. To this end, using UBCG as a reference appears to be more convenient, as once these core genes have been extracted, if a new species needed to be added to the database, only the core genes for that species have to be calculated.

#### Evaluating the Value of Genomospecies References in a Metagenomic Database

To assess the effects of adding genomospecies to the reference database, we classified the same samples with two custom pipelines, one with only valid named *Streptococcus* species (KrakenUBCG-VNS), and a second one with species and genomospecies (KrakenUBCG), the rest of the bacterial references are identical, as described in Table 1. Additionally, we also ran the classifications using different cutoff values for Kraken. These cutoff values represent the k-mer coverage required for a read to be considered classified. A cutoff value of 0 means that only one k-mer is required for the read to be classified, while a cutoff value of 1 requires that the entirety of the read needs to be covered by *N* number of k-mers belonging to the database, where *N* = read size – k + 1, in other words, an exact match. 

**Figure 6:**
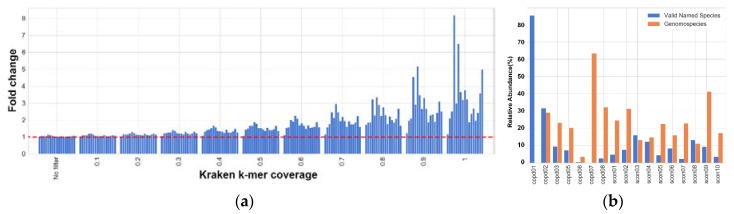
(**a**) Fold change for k-mer coverage thresholds that the KrakenUBCG pipeline has against KrakenUBCG-VNS, illustrating that with higher thresholds, higher read classification rate is achieved by the genomospecies database. (**b**) Abundance predicted by KrakenUBCG, separating the abundance assigned to valid species and genomospecies from *Streptococcus* (genus).

**Figure 7:**
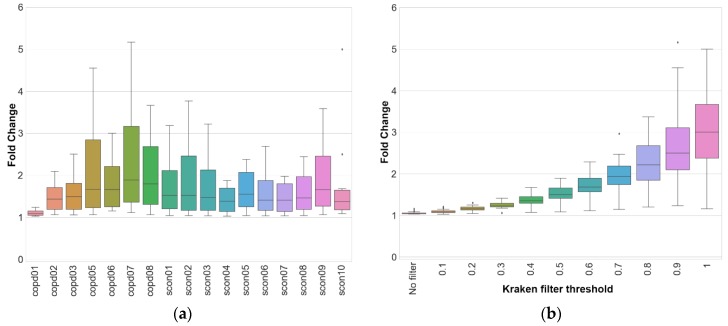
(**a**) Range and median fold change per sample for all the different k-mer coverage thresholds. Samples with high abundance ratio of genomospecies had the highest median fold change against the database with just valid species (KrakenUBCG-VNS). (**b**) Range and median fold change for several identity thresholds when comparing KrakenUBCG with KrakenUBCG-VNS, showing that with higher identity thresholds, higher classification rate is achieved by the genomospecies pipeline.

Figure 6a shows the fold change of classified reads for each sample at each cutoff value. The fold change represents the ratio of number of reads classified by KrakenUBCG against the number of reads classified by KrakenUBCG-VNS. At a cutoff value of 0 (no filter), the fold change of classified reads is closer to 1 (median value of 1.04), meaning that either pipeline (with or without genomospecies) will classify almost the same number of reads at the genus level, however at higher cutoff values, the fold change of classified reads will increase greatly when using the genomospecies database KrakenUBCG. Just with a cutoff value of 0.3, the median fold change is 1.227, this means that having genomospecies references present in the reference database will classify more than 22% additional reads. A stricter cut off value of 0.7 will have a median fold change of 1.935, resulting in 93% additional classified reads when comparing with the database without genomospecies KrakenUBCG-VNS. As expected, including genomospecies references not only increased the number of reads classified for the genus *Streptococcus*, but also by increasing the cutoff value of Kraken allowed us to see how closer the references are to the reads in the samples. The argument can be made that, inclusion of additional references to any database would incur in a higher number of classified reads, however, when comparing the fold change for each sample, we can see that this is not always the case. As seen previously in Figure 4, sample copd01 only contains the valid named species *Streptococcus pneumoniae* and *Streptococcus peroris*, and this prediction can be confirmed by the low fold change (Figure 7a) between pipelines (with or without genomospecies) and their different cutoff values (median fold change of 1.076). The rest of the samples showed the presence of at least one genomospecies, explaining the median fold difference between classifications (median range between 1.37 and 1.89). Sample copd07 showed the highest median fold change among all samples (median of 1.89), this is explained by Figure 4, showing that this sample contains 5 genomospecies without containing a single validly named species of *Streptococcus*.

These observed fold changes between classifications and distinct cut off values can only be explained if these samples indeed contain one or more genomospecies in their sample. Not including these genomospecies will result in the loss of classified reads and misidentification at species level. Samples that do not contain any genomospecies showed no significant change between databases and cutoff values. In addition, by using the same 92 core genes as our reference for every species, it allows us to discard any potential contamination or bias in our analysis based on genome length, assembly status, or incompleteness of the genome. All species and genomospecies are represented equally in our databases (92 core genes per reference, one reference per species), so any additional classified reads can only be explained by the presence or absence of the reference that matches closely with the reads within the samples.

Figure 7b shows the fold difference between the total number of reads classified between the KrakenUBCG and KrakenUBCG-VNS. When the classification thresholds are removed (no filter), both pipelines are able to classify almost the same number of reads, since only one k-mer match is necessary to label a read as classified. When this identity threshold is increased gradually, the number of reads classified with KrakenUBCG-VNS decrease at a higher rate when comparing with KrakenUBCG, concluding that a majority of the reads have higher coverage with most of these genomospecies references. At an identity threshold of 0.8, KrakenUBCG is able to classify more than 2-fold the amount of reads.

### 2.4. Identifying Accurately a Streptococcal Infection Using Clinical Data

Tracing the origin of pathogenic bacteria in bloodstream infections can be a challenge. Tamburini et al. [16] proposed that the source of infection on some patients is the human gut. They isolated and sequenced pathogenic bacteria present in the bloodstream of patients at Stanford University Hospital and performed shotgun metagenomics on stool samples for those same patients. For this research, we will focus on patient 22, whereby using his blood isolate and stool sample, the authors were able to match the genome assembled from the bloodstream isolate with the metagenomic assembly generated form the stool sample and classified it as *Streptococcus mitis*.

We analyzed the streptococcal bacterial isolate from this patient (Sample id: SRR7407865) using the TrueBac ID system [17], which utilizes the exact same species and genomospecies references from the EzBioCloud database included in our KrakenUBCG pipeline. Table 2 shows the results of TrueBac ID when comparing the isolate with the EzBioCloud database. Our analysis indicates that the closest reference to the isolate is actually streptococcal genomospecies JPFV_s (not *Streptococcus mitis)* with an ANI identity value of 94.61% with a coverage of 86.9%. Since the ANI identity value is below the species threshold (95%) [18], we will assume that this isolate belongs to a tentative new genomospecies and does not belong to *Streptococcus mitis*.

To further illustrates the utility of broader species coverage and need to update the reference databases frequently, we built a third metagenomic reference database (KrakenUBCG+ SRR7407865) with the addition of this tentative new genomospecies by assembling the genome and extracting the 92 core genes using the UBCG pipeline and simply adding them to the KrakenUBCG database. We then perform a metagenome classification analysis using all three databases.

**Figure 8:**
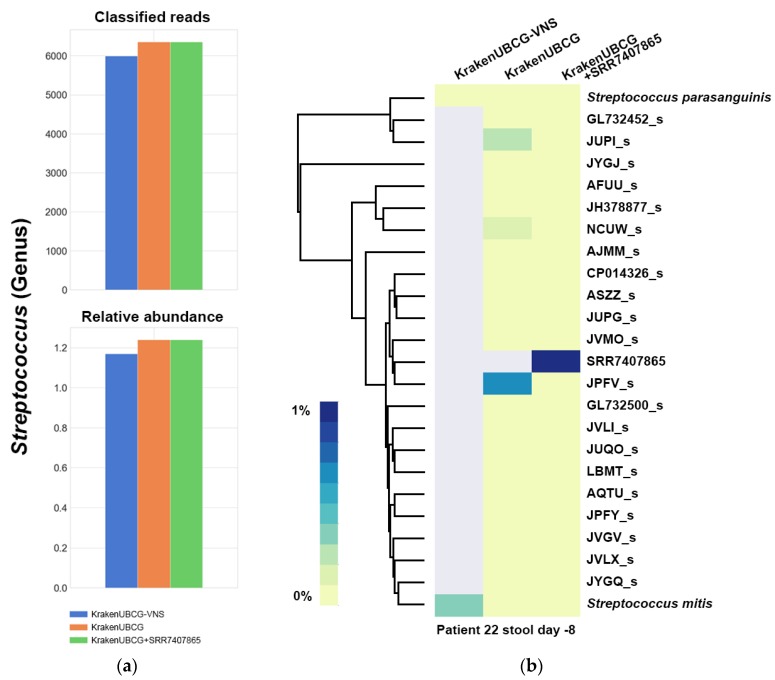
(**a**) Number of classified reads and relative abundance at genus level for the stool sample of patient 22. (**b**) Taxonomy classification of *Streptococcus* for the three pipelines containing different references.

**Figure 9:**
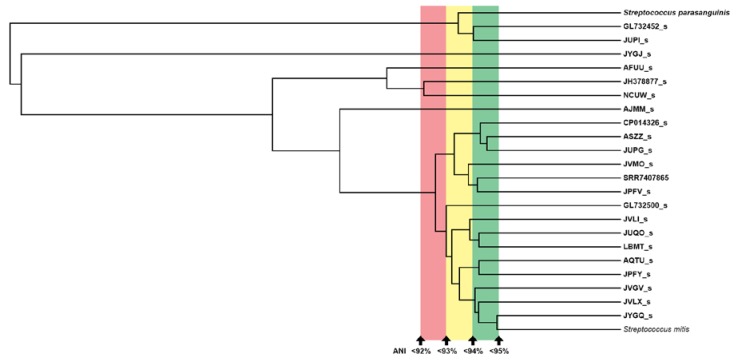
Average nucleotide identity (ANI) dendrogram with different ANI thresholds highlighted for the sample of patient 22, showing distinct possible genomospecies for this streptococcal subtree.

Figure 8 shows the classification results for all the available pipelines. Relative abundance at the genus level for *Streptococcus* yields an increase of 0.07% after classifying with KrakenUBCG, while it only showed an increase of 0.002% when adding the bloodstream isolate from the sample (KrakenUBCG+ SRR7407865). However, at the species level, the classifications for all three databases showed big differences. For the classification with only valid named species (KrakenUBCG-VNS), the highest, most abundant species of *Streptococcus* is *Streptococcus mitis*, however by using KrakenUBCG, the most abundant species is JPFV_s. By observing those two different classifications using the ANI tree shown in Figure 9, we can observe that they are both correct, with the absence of references from streptococcal genomospecies, the classification should go to the closest reference, in this case, *Streptococcus mitis*. This is also consistent with the initial classification of the isolate in the original paper as *Streptococcus mitis* [16].

Finally, the third classification using KrakenUBCG+ SRR7407865, showed that by comparing the stool sample with an updated database that contains a bloodstream isolate, will successfully assign the top streptococcal species as this tentative novel genomospecies. 

While for this case, we had access to the bacterial blood isolate, the majority of the time, the user will have to rely on precompiled databases, and in this specific case, if we were to use our genomospecies database, the classification for this streptococcal species would be assigned to the closest reference available in the database, in this case, the genomospecies JPFV_s. We also show that in the absence of any genomospecies in the reference database, this streptococcal strain would be classified as *Streptococcus mitis*. This demonstrates the importance of updating reference databases frequently, even if those references have not been published or named formally.

### 2.5. Effects of Different ANI Thresholds on the Classificwtion of Genomospecies

The ANI threshold for genomospecies identification proposed by Chun et al. [18] is 95%, and this threshold is applied at the EzBioCloud database. As the authors mention in their article, these are minimal standards, which means that in some cases, a different ANI threshold could be applied in order to set species boundaries for a specific taxon. Similarly, the Genome Taxonomic Database (GTDB) [19] also proposes a 95% ANI threshold for these novel species using FastANI [20], although using a different nomenclature for these genomospecies, supporting this ANI threshold as a consensus in the prokaryote taxonomic community. In the previous example shown in Figure 8, the blood isolate from patient 22 was recognized as a potential new species because the ANI value between the isolate and the closest genomospecies JPFV_s is 94.24%, which is below the proposed species boundary. However, if a different ANI threshold was set, for example 94%, this blood isolate would be considered as a part of the genomospecies JPFV. With this in mind, we decided to change the ANI threshold for genomospecies for the case of patient 22 in order to see the effects that a different ANI threshold would have on the classification of streptococcal bacteria present in the stool sample. Figure 9 shows the ANI dendrogram marked with the different ANI thresholds used in this analysis.

**Figure 10:**
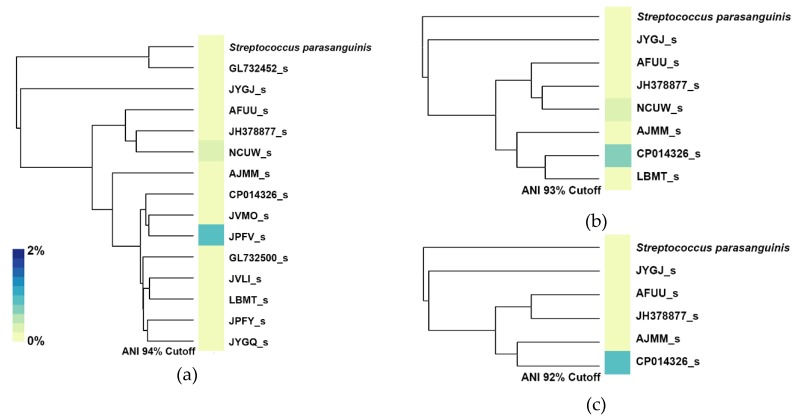
Classification of streptococcal species using different ANI thresholds for genomospecies boundaries, (**a**) 94%, (**b**) 93%, and (**c**) 92%, showing the changes of species detection depending on the ANI threshold used.

For this experiment, we built three additional databases, each one containing a different number of streptococcal genomospecies depending on the ANI threshold set for each database. When setting a new ANI threshold, only one reference would be used as a reference for that genomospecies. For example, at an ANI threshold of 94%, the genomospecies JUQO_s and LBMT_s would be considered as a single genomospecies, so in order to select which reference would be used to represent this new genomospecies, we selected the genome with the highest N50 value. After building these three additional databases, we profiled the stool sample of patient 22 and generated heatmaps with their respective ANI dendrogram as shown in Figure 10. With a 94% ANI cutoff, the blood isolate from patient 22 is now considered as the JPFV_s genomospecies, at the same time, JUPI_s now is considered a strain of GL732452_s; despite these changes, the main streptococcal species found on the sample is JPFV_s, which is the genomospecies that now represents the blood isolate from the patient. A small abundance is also detected for the genomospecies NCUW_s. Lowering the ANI threshold to 93%, decreases the number of genomospecies present in this tree, with CP014326_s now representing a vast majority of genomospecies from the 95% ANI tree, including ASZZ_s, JUPG_s, JVMO_s, JPFV_s, and the blood isolate SRR7407865. In this case, CP014326_s is classified as the main streptococcal species with NCUW_s also being found present with a low abundance. Lastly, with an ANI threshold of 92%, only six species remain, this time, the genomospecies CP014326_s maintains its predominant presence of streptococcal abundance, with a slight abundance increment, and this could be mainly due to the absence of NCUW_s, now being represented by JH378877_s.

The example shown in Figure 10, demonstrated that regardless of the ANI threshold set for genomospecies, the detection of a specific species can be achieved if a representative species close to the one of interest is present in the database. With an ANI threshold of 92%, genomospecies CP014326_s represents our blood isolate SRR7407865, and with an ANI distance between these two of 93.591%, it can be seen that with a small sacrifice of abundance detection, the presence of either genomospecies can be achieved regardless of the ANI threshold set by the user.

Here, we showed that even with different ANI thresholds, the classification of the streptococcal strain was consistent with the location of the blood isolate within the original ANI dendrogram. However, a loss of abundance was seen mainly because of the higher ANI distance between the species present in the stool sample and the reference used to represent the genomospecies on each of the examples. While the ANI threshold for setting boundaries for new species can be debatable, when using a computational method that is based on sequence similarity, we believe that a fixed sequence similarity ANI threshold should be implemented, regardless of the taxonomic rank we set to given a taxon node (species, genomospecies, or even strain).

## 3. Materials and Methods 

### 3.1. Selecting the Reference Genomes

We used the EzBioCloud database [8] as a source for our reference genomes and selected one reference per species with a total of 9145 bacterial species, including 201 species (88 validly named species and 113 genomospecies) in the genus *Streptococcus*. Supplementary Table S1 shows the list of references used, with their respective EzBioCloud and NCBI accession numbers. Supplementary Figure S1 shows the ANI dendrogram of all the streptococcal references used in this study. For Section 2.4, we merged taxonomic nodes depending on the new ANI threshold set by selecting only one reference with the highest N50 value for their assembly; N50 is defined as the length of the shortest contig that accumulatively show 50% or more of the genome size [18]. 

The genome sequence of a blood isolate from patient 22 (NCBI accession SRR7407865) was incorporated to the database for Section 2.3. The raw data was downloaded from NCBI and assembled using the pipeline SPAdes [21] with the default parameters.

### 3.2. Average Nuclodetide Identity and Hierarchical Clustering

OrthoANIu [22] was used to calculate ANI. Hierarchical clustering was carried out from the ANI matrix by applying the UPGMA (unweighted pair group method with arithmetic mean) algorithm using the R library phangorn [23].

### 3.3. Synthetic and Real Metagenomic Samples

The four simulated datasets used to compare our pipeline with MetaPhlAn2 were generated with InSilicoSeq [13] using the MiSeq model provided. These datasets are available at https://bitbucket.org/streptosynth/metagenome/downloads/. Supplementary Table S2 shows the genomes used for these sets. The chronic obstructive pulmonary disease metagenomic samples [14] were downloaded from https://www.ncbi.nlm.nih.gov/bioproject/PRJEB9034/, while the metagenome and isolate samples of the patient 22 [16] was from https://www.ncbi.nlm.nih.gov/bioproject/PRJNA477326/. 

### 3.4. Extracting the Core Genes

**Figure 11:**
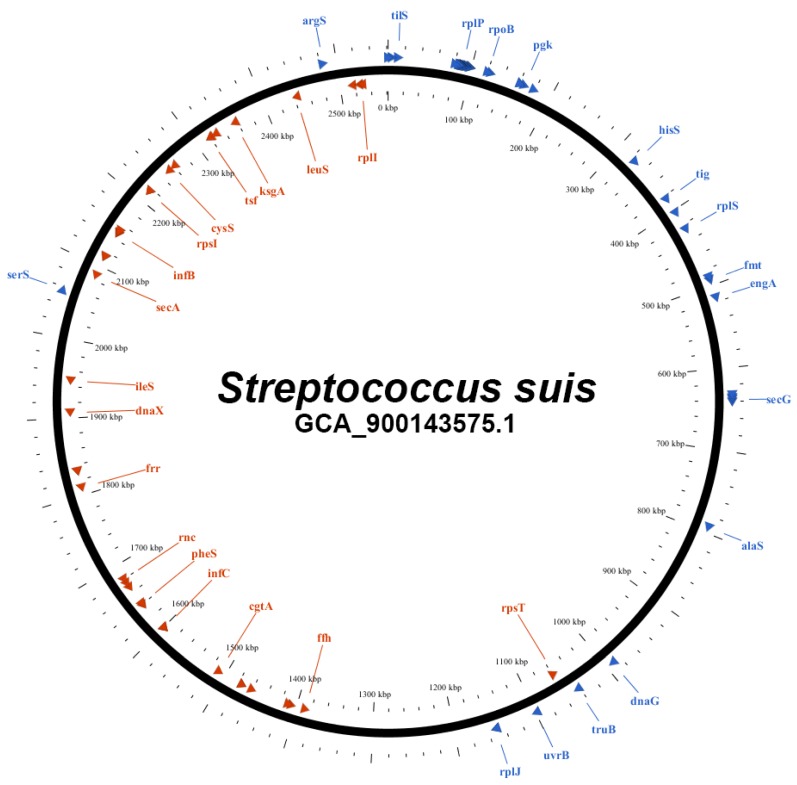
Location of the 92 core genes on the reference for *Streptococcus suis*.

The 92 genes that are defined as up-to-date bacterial core gene (UBCG) were extracted for all species from EzBioCloud databases, including those belonging to the genus *Streptococcus* as described earlier [2]. Figure 11 shows the distribution and location of all 92 UBCG of the *Streptococcus suis* reference genome (NCBI accession GCA_900143575.1). All 92 UBCGs were also extracted from the genome assembly of a blood isolate (NCBI SRA accession SRR7407865).

### 3.5. Taxonomic Profiling

The publicly available software Kraken was used to build the UBCG k-mer database. Kraken is a k-mer based metagenomic taxonomic classifier that utilizes a modified HashMap created by Jellyfish [24] in order to classify sequences with higher speeds and accuracy. First, using EzBioCloud’s taxonomy, we created a taxonomic structure compatible with Kraken and then we converted all the UBCG references to fasta format. Once the references were added, we compiled the database with a k-mer size of 26 bp. A normalization database using Bracken [25] was also generated for different read lengths (100, 200, 250). Bracken distributes reads classified at higher taxonomic levels to a species level classification.

In this study, a total of 5 databases using UBCGs were built, one containing just validly named species (KrakenUBCG-VNS), a second one containing the same valid named species with the addition of genomospecies references (KrakenUBCG) defined by the ANI threshold of 95% proposed by Chun et al. [18]. Lastly, three databases were build using different ANI thresholds (94%, 93%, and 92%). For each of these databases, we maintained the ‘one reference per taxon’ approach, and the method of selecting the representative genome for these databases can be found in Section 4.

To classify the metagenomes using Kraken, all samples were classified with a Kraken filter threshold of 0.1 with the exception of Section 2.2 where different thresholds were used to demonstrate closeness of the reference with the metagenomic reads. Kraken’s threshold per read is calculated as the fraction C/Q, where C is the number of k-mers mapped to the lowest common ancestor values in the clade rooted at the label, and Q is the number of k-mers in the sequence that lack an ambiguous nucleotide.

After the classification by Kraken and the normalization step by Bracken, all metagenomic abundances predicted with our UBCG database were normalized using the total length of all the UBCG belonging to a single species.

To compare our results, we also predicted the abundances at species level using MetaPhlAn2 [1] with the v20 version of database and default parameters. In order to compare fairly our predictions with MetaPhlAn2, we matched their reference markers using the NCBI accession number provided in their database and EzBioCloud’s NCBI’s accession numbers and matched them when present in their database as seen in Figure 4. Abundance accuracy for the synthetic datasets was measured using the log-modulus difference between the truth and predicted abundances of both pipelines [26]. The log-modulus was calculated as:

y’ = sign(y) × log10(1 + |y|),(1)

to preserve the sign of the difference between estimated and expected abundance, y. 

### 3.6. Biomarker Discovery

Biomarker discovery shown in Figure 5 was calculated using LEfSe [15], which utilizes a linear discriminant analysis (LDA) effect size method to support high-dimensional class comparisons. LEfSe’s biomarkers were found using a P-value <0.05 (Kruskal–Wallis test) and an LDA score (log 10) > 2.0.

## 4. Conclusions

In this study, we demonstrated that UBCG sequences can be used as references for metagenomic classification, showing that they are easy to extract from genome sequences and accurate when predicting relative abundance. However, UBCG sequences can only represent bacterial organisms, so in order to represent other type of organisms, a different set of core genes needs to be used. We also demonstrated that inclusion of the genomospecies in the reference databases significantly improve the classification accuracy of bacterial species within a metagenomic sample. Furthermore, our study implicated that, regardless of the ANI threshold used for species/genomospecies boundaries, if the genomic reference sequence does not change, only the name for the given species will be different. Finally, we showed that while publicly available pipelines and databases are easily accessible, for accurate and reliable taxonomic classification, an updated database with proper taxonomic and genomic curation must be used.

## Figures and Tables

**Table 1:** Comparison of taxonomic profiling pipelines used in this study.

Pipeline	Algorithm	Database	Gene Type Included in the Database	Number of Bacterial Species	Number of *Streptococcus* Species
KrakenUBCG	Exact match k-mer	EzBioCloud	Bacterial Core Genes (92 UBCG per species)	14,272 species ( 9145 validly named+ 5127 genomospecies)	201 species (88 validly named+ 113 genomospecies)
KrakenUBCG-VNS	Exact match k-mer	EzBioCloud	Bacterial Core Genes (92 UBCG per species)	9145 validly named species	88 validly named species
MetaPhlAn2	Burrows–Wheeler	NCBI	Marker Genes (variable number)	3310 species ( 2250 validly named+ 1060 unidentified species)	72 species (53 validly named+ 19 unnamed genomospecies)

**Table 2:** TrueBac ID analysis of the *Streptococcus* isolate from the bloodstream of patient 22.

No.	Hit Taxon	ANI (%)	ANI Coverage (%)	16S (%)	recA (%)	rplC (%)
1	JPFV_s	94.61	86.9	99.93	94.86	99.36
2	JVMO_s	94.34	85.8	99.59	95.72	98.88
3	JPFU_s	93.06	74.9	99.59	94.55	99.20
4	JUUO_s	93.44	78.5	99.39	94.60	99.68
5	JYGP_s	93.40	75.3	99.18	94.77	99.20

## Supplementary Materials

The following are available online at https://www.mdpi.com/2076-0817/9/3/204/s1, Figure S1: ANI phylogenetic tree of all the streptococcal references used in this study, Table S1: Table containing the names and accession numbers for every streptococcal genomic reference used in this study, Table S2: Table containing the references used on the synthetic datasets, containing also the true abundances for each sample, Table S3: Table containing the streptococcal references contained on MetaPhlAn2.

## References

[1] Truong D.T., Franzosa E.A., Tickle T.L., Scholz M., Weingart G., Pasolli E., Tett A., Huttenhower C., Segata N. (2015). MetaPhlAn2 for enhanced metagenomic taxonomic profiling. Nat. Methods.

[2] Na S., Kim Y.O., Yoon S., Ha S., Baek I., Chun J. (2018). UBCG: Up-to-date bacterial core gene set and pipeline for phylogenomic tree reconstruction §. J. Microbiol..

[3] Gao X.-Y., Zhi X.-Y., Li H.-W., Klenk H.-P., Li W.-J. (2014). Comparative Genomics of the Bacterial Genus Streptococcus Illuminates Evolutionary Implications of Species Groups. PLoS ONE.

[4] Linares D.M., O’Callaghan T.F., O’Connor P.M., Ross R.P., Stanton C. (2016). Streptococcus thermophilus APC151 Strain Is Suitable for the Manufacture of Naturally GABA-Enriched Bioactive Yogurt. Front. Microbiol..

[5] Krzyściak W., Pluskwa K.K., Jurczak A., Kościelniak D. (2013). The pathogenicity of the Streptococcus genus. Eur. J. Clin. Microbiol. Infect. Dis..

[6] Shelburne S., Sahasrabhojane P., Saldaña M., Yao H., Su X., Horstmann N., Thompson E., Flores A.R. (2014). Streptococcus mitis Strains Causing Severe Clinical Disease in Cancer Patients. Emerg. Infect. Dis..

[7] Ehara N., Fukushima K., Kakeya H., Mukae H., Akamatsu S., Kageyama A., Saito A., Kohno S. (2008). A novel method for rapid detection of Streptococcus pneumoniae antigen in sputum and its application in adult respiratory tract infections. J. Med. Microbiol..

[8] Yoon S.-H., Ha S.-M., Kwon S., Lim J., Kim Y., Seo H., Chun J. (2017). Introducing EzBioCloud: A taxonomically united database of 16S rRNA gene sequences and whole-genome assemblies. Int. J. Syst. Evol. Microbiol..

[9] Patil P.P., Kumar S., Midha S., Gautam V., Patil P.B. (2018). Taxonogenomics reveal multiple novel genomospecies associated with clinical isolates of Stenotrophomonas maltophilia. Microb. Genom..

[10] Fischer S., Mayer-Scholl A., Imholt C., Spierling N.G., Heuser E., Schmidt S., Reil D., Rosenfeld U.M., Jacob J., Nöckler K. (2018). Leptospira Genomospecies and Sequence Type Prevalence in Small Mammal Populations in Germany. Vector Borne Zoonotic Dis..

[11] Salipante S.J., Kalapila A., Pottinger P.S., Hoogestraat D.R., Cummings L., Duchin J.S., Sengupta D.J., Pergam S.A., Cookson B.T., Butler-Wu S.M. (2015). Characterization of a Multidrug-Resistant, Novel Bacteroides Genomospecies. Emerg. Infect. Dis..

[12] Wood D.E., Salzberg S. (2014). Kraken: Ultrafast metagenomic sequence classification using exact alignments. Genome Boil..

[13] Karlsson-lindsjo O., Hayer J., Bongcam-rudloff E. (2019). Sequence analysis Simulating Illumina metagenomic data with InSilicoSeq. Bioinformatics.

[14] Cameron S., Lewis K., Huws S.A., Lin W., Hegarty M.J., Lewis P.D., Mur L.A.J., Pachebat J. (2016). Metagenomic Sequencing of the Chronic Obstructive Pulmonary Disease Upper Bronchial Tract Microbiome Reveals Functional Changes Associated with Disease Severity. PLoS ONE.

[15] Segata N., Izard J., Waldron L., Gevers D., Miropolsky L., Garrett W.S., Huttenhower C. (2011). Metagenomic biomarker discovery and explanation. Genome Biol..

[16] Tamburini F.B., Andermann T.M., Tkachenko E., Senchyna F., Banaei N., Bhatt A.S. (2018). Precision identification of diverse bloodstream pathogens in the gut microbiome. Nat. Med..

[17] Ha S.-M., Kim C.K., Roh J., Byun J.-H., Yang S.-J., Choi S.-B., Chun J., Yong J. (2019). Application of the Whole Genome-Based Bacterial Identification System, TrueBac ID, Using Clinical Isolates That Were Not Identified With Three Matrix-Assisted Laser Desorption/Ionization Time-of-Flight Mass Spectrometry (MALDI-TOF MS) Systems. Ann. Lab. Med..

[18] Chun J., Oren A., Ventosa A., Christensen H., Arahal D.R., Da Costa M., Rooney A., Yi H., Xu X.-W., De Meyer S. (2018). Proposed minimal standards for the use of genome data for the taxonomy of prokaryotes. Int. J. Syst. Evol. Microbiol..

[19] Chaumeil P.-A., Mussig A.J., Hugenholtz P., Parks D.H. (2019). GTDB-Tk: A toolkit to classify genomes with the Genome Taxonomy Database. https://academic.oup.com/bioinformatics/advance-article/doi/10.1093/bioinformatics/btz848/5626182.

[20] Jain C., Rodriguez-R L.M., Phillippy A.M., Konstantinidis K.T., Aluru S. (2018). High throughput ANI analysis of 90K prokaryotic genomes reveals clear species boundaries. Nat. Commun..

[21] Bankevich A., Nurk S., Antipov D., Gurevich A.A., Dvorkin M., Kulikov A.S., Lesin V.M., Nikolenko S.I., Pham S., Prjibelski A.D. (2012). SPAdes: A new genome assembly algorithm and its applications to single-cell sequencing. J. Comput. Biol..

[22] Yoon S.-H., Lim J., Kwon S., Ha S.-M., Chun J. (2017). A large-scale evaluation of algorithms to calculate average nucleotide identity. Antonie van Leeuwenhoek.

[23] Schliep K.P. (2011). Phangorn: Phylogenetic analysis in R. Bioinformatics.

[24] Marçais G., Kingsford C. (2011). A fast, lock-free approach for efficient parallel counting of occurrences of k-mers. Bioinformatics.

[25] Lu J., Breitwieser F.P., Thielen P., Salzberg S. (2017). Bracken: Estimating species abundance in metagenomics data. PeerJ Comput. Sci..

[26] McIntyre A., Ounit R., Afshinnekoo E., Prill R.J., Henaff E., Alexander N., Minot S.S., Danko D., Foox J., Ahsanuddin S. (2017). Comprehensive benchmarking and ensemble approaches for metagenomic classifiers. Genome Biol..

