# Supplementary material for: Improved Metagenomic Taxonomic Profiling Using a Curated Core Gene-Based Bacterial Database Reveals Unrecognized Species in the Genus Streptococcus
Source: Pathogens. 2020 Mar 10;9(3):204. doi: 10.3390/pathogens9030204 (PMC7157611; doi:10.3390/pathogens9030204)
Supplement: Supplementary file 1 [file pathogens-09-00204-s001.zip › Supplementary-Tables.pdf]

Table S1. Species from the genus streptococcus covered in KrakenUBCG.

NCBI Accession	NCBI name	Strain name	EzBioCloud name	Genome Size
GCA_000188295.1	Streptococcus vestibularis ATCC 49124	ATCC 49124	Streptococcus vestibularis	1872773
GCA_001375655.1	Streptococcus varani	FF10	Streptococcus varani	2460376
GCA_000188055.3	Streptococcus urinalis 2285-97	2285-97	Streptococcus urinalis	2130431
GCA_900475595.1	Streptococcus uberis	NCTC 3858	Streptococcus uberis	1975601
GCA_002355215.1	Streptococcus troglodytae	TKU 31	Streptococcus troglodytae	2097874
GCA_900095845.1	Streptococcus timonensis	Marseille-P2915	Streptococcus timonensis	1925331
GCA_000380145.1	Streptococcus thoraltensis DSM 12221	DSM 12221	Streptococcus thoraltensis	2385218
GCA_900474985.1	Streptococcus thermophilus	NCTC 12958	Streptococcus thermophilus	2102271
GCA_900475585.1	Streptococcus suis	NCTC 10234	Streptococcus suis	1984375
GCA_900475395.1	Streptococcus sobrinus	NCTC 12279	Streptococcus sobrinus	2198648
GCA_000767835.1	Streptococcus sinensis	HKU4	Streptococcus sinensis	2061843
GCA_900475505.1	Streptococcus sanguinis	NCTC 7863	Streptococcus sanguinis	2296927
GCA_000253335.1	Streptococcus salivarius CCHSS3	JIM8780	Streptococcus salivarius subsp. salivarius	2217184
GCA_003609975.1	Streptococcus ruminantium	GUT-187	Streptococcus ruminantium	2090539
GCA_003595525.1	Streptococcus respiraculi	HTS25	Streptococcus respiraculi	2067971
GCA_000286075.1	Streptococcus ratti FA-1 = DSM 20564	FA-1	Streptococcus ratti	2062017
GCA_002055535.1	Streptococcus pyogenes	NCTC 8198	Streptococcus pyogenes	1914862
GCA_000188035.3	Streptococcus pseudoporcinus LQ 940-04	LQ 940-04	Streptococcus pseudoporcinus	2126865
GCA_002087075.1	[illegible]tococcus pseudopneumoniae ATCC BAA-960 = CCUG 4[illegible]	CCUG 49455	Streptococcus pseudopneumoniae	2172620
GCA_900475415.1	Streptococcus porcinus	NCTC 10999	Streptococcus porcinus	2033046
GCA_000423765.1	Streptococcus porci DSM 23759	DSM 23759	Streptococcus porci	2290936
GCA_001457635.1	Streptococcus pneumoniae	NCTC 7465	Streptococcus pneumoniae	2110968
GCA_000423745.1	Streptococcus plurextorum DSM 22810	DSM 22810	Streptococcus plurextorum	2103464
GCA_002953735.1	Streptococcus pluranimalium	TH11417	Streptococcus pluranimalium	2065522
GCA_000772915.1	Streptococcus phocae C-4	C-4	Streptococcus phocae subsp. salmonis	1659203
GCA_001302265.1	Streptococcus phocae	ATCC 51973	Streptococcus phocae subsp. phocae	1700082
GCA_000187585.1	Streptococcus peroris ATCC 700780	ATCC 700780	Streptococcus peroris	1639920
GCA_002887775.1	Streptococcus sp. CAIM 1838	CAIM 1838	Streptococcus penaeicida	2051969
GCA_000187935.2	Streptococcus parauberis NCFD 2020	NCFD 2020	Streptococcus parauberis	2164480
GCA_000440555.1	Streptococcus suis 86-5192	86-5192	Streptococcus parasuis	2110166
GCA_000164675.2	Streptococcus parasanguinis ATCC 15912	ATCC 15912	Streptococcus parasanguinis	2153652
GCA_001642085.1	Streptococcus pantholopis	TA 26	Streptococcus pantholopis	2241302
GCA_000380125.1	Streptococcus ovis DSM 16829	DSM 16829	Streptococcus ovis	2358538
GCA_000380105.1	Streptococcus orisratti DSM 15617	DSM 15617	Streptococcus orisratti	2416134
GCA_002093515.1	Streptococcus oralis subsp. tigurinus	AZ_14	Streptococcus oralis subsp. tigurinus	1964273
GCA_000164095.1	Streptococcus oralis ATCC 35037	ATCC 35037	Streptococcus oralis subsp. oralis	1913838
GCA_000382825.1	Streptococcus dentisani 7747	CECT 7747	Streptococcus oralis subsp. dentisani	1884389
GCA_900475095.1	Streptococcus mutans	NCTC 10449	Streptococcus mutans	2019343

GCA_000148585.1	Streptococcus mitis NCTC 12261	NCTC 12261	Streptococcus mitis	1831081
GCA_000377005.1	Streptococcus minor DSM 17118	DSM 17118	Streptococcus minor	1927889
GCA_900187085.1	Streptococcus merionis	NCTC 13788	Streptococcus merionis	2384128
GCA_900459365.1	Streptococcus massiliensis	NCTC 13765	Streptococcus massiliensis	2033868
GCA_001623565.1	Streptococcus sp. HTS5	HTS5	Streptococcus marmotae	2322791
GCA_000380045.1	Streptococcus marimammalium DSM 18627	DSM 18627	Streptococcus marimammalium	1505444
GCA_000187995.3	Streptococcus macacae NCTC 11558	NCTC 11558	Streptococcus macacae	1916985
GCA_900475675.1	Streptococcus lutetiensis	NCTC 13774	Streptococcus lutetiensis	1793521
GCA_900475975.1	Streptococcus intermedius	NCTC 11324	Streptococcus intermedius	1932951
GCA_001595425.1	Streptococcus iniae	CAIM 527	Streptococcus iniae	2103840
GCA_000187465.1	Streptococcus infantis ATCC 700779	ATCC 700779	Streptococcus infantis	1905984
GCA_900459445.1	Streptococcus infantarius	NCTC 13760	Streptococcus infantarius	1974692
GCA_000188015.3	Streptococcus ictaluri 707-05	707-05	Streptococcus ictaluri	2234402
GCA_000420785.1	Streptococcus hyovaginalis DSM 12219	DSM 12219	Streptococcus hyovaginalis	2077809
GCA_900459405.1	Streptococcus hyointestinalis	NCTC 12224	Streptococcus hyointestinalis	2553358
GCA_000785785.1	Streptococcus uberis	CAIM 1894	Streptococcus hongkongensis	2149440
GCA_001708305.1	Streptococcus himalayensis	HTS2	Streptococcus himalayensis	2275471
GCA_000376985.1	Streptococcus henryi DSM 19005	DSM 19005	Streptococcus henryi	2422509
GCA_001598035.1	Streptococcus sp. HTS9	HTS9	Streptococcus halotolerans	2182100
GCA_900475015.1	Streptococcus gordonii	NCTC 7865	Streptococcus gordonii	2187611
GCA_900478025.1	Streptococcus pasteurianus	NCTC 13784	Streptococcus gallolyticus subsp. pasteurianus	2178089
GCA_900459545.1	Streptococcus gallolyticus	NCTC 13767	Streptococcus gallolyticus subsp. macedonicus	2312921
GCA_900475715.1	Streptococcus gallolyticus	NCTC 13773	Streptococcus gallolyticus subsp. gallolyticus	2493581
GCA_900475025.1	Streptococcus ferus	NCTC 12278	Streptococcus ferus	1872314
GCA_900459295.1	Streptococcus equinus	NCTC 12969	Streptococcus equinus	1806069
GCA_900459475.1	Streptococcus equi subsp. zooepidemicus	NCTC 4676	Streptococcus equi subsp. zooepidemicus	2109071
GCA_000706805.1	Streptococcus equi subsp. ruminatorum CECT 5772	CECT 5772	Streptococcus equi subsp. ruminatorum	2138288
GCA_900156215.1	Streptococcus equi	ATCC 33398	Streptococcus equi subsp. equi	2137632
GCA_000380025.1	Streptococcus entericus DSM 14446	DSM 14446	Streptococcus entericus	2036468
GCA_900459095.1	Streptococcus dysgalactiae	NCTC 13762	Streptococcus dysgalactiae subsp. equisimilis	2285205
GCA_900459225.1	Streptococcus dysgalactiae subsp. dysgalactiae	NCTC 13731	Streptococcus dysgalactiae subsp. dysgalactiae	2151704
GCA_900459175.1	Streptococcus downei MFe28	NCTC 11391	Streptococcus downei	2232110
GCA_000380005.1	Streptococcus didelphis DSM 15616	DSM 15616	Streptococcus didelphis	1877438
GCA_000423725.1	Streptococcus devriesei DSM 19639	DSM 19639	Streptococcus devriesei	2121372
GCA_001921845.1	Streptococcus cuniculi	NED12-00049-6B	Streptococcus cuniculi	2094853
GCA_900475445.1	Streptococcus cristatus ATCC 51100	NCTC 12479	Streptococcus cristatus	2000351
GCA_000187975.3	Streptococcus criceti HS-6	HS-6	Streptococcus criceti	2418558
GCA_000463425.1	Streptococcus constellatus subsp. pharyngis C1050	C1050	Streptococcus constellatus subsp. pharyngis	1991156
GCA_900459125.1	Streptococcus constellatus	NCTC 11325	Streptococcus constellatus subsp. constellatus	1906855
GCA_003086355.2	Streptococcus sp. Z15	Z15	Streptococcus chenjunshii	2443046
GCA_001937065.1	Streptococcus sp. 'caviae'	Cavy grass 6	Streptococcus caviae	2108609

GCA_000425025.1	Streptococcus castoreus DSM 17536	DSM 17536	Streptococcus castoreus	1883536
GCA_000268305.2	Streptococcus canis FSL Z3-227	FSL Z3-227	Streptococcus canis	2267856
GCA_000379985.1	Streptococcus caballi DSM 19004	DSM 19004	Streptococcus caballi	2122044
GCA_001984715.1	Streptococcus azizii	Dec-02	Streptococcus azizii	2340839
GCA_900476055.1	Streptococcus australis	NCTC 13166	Streptococcus australis	2161464
GCA_000257765.1	Streptococcus anginosus subsp. whileyi CCUG 39159	CCUG 39159	Streptococcus anginosus subsp. whileyi	2294730
GCA_000463465.1	Streptococcus anginosus C1051	C1051	Streptococcus anginosus subsp. anginosus	1911706
GCA_900458965.1	Streptococcus agalactiae	NCTC 8181	Streptococcus agalactiae	2448053
GCA_900459045.1	Streptococcus acidominimus	NCTC 12957	Streptococcus acidominimus	2445682
GCA_003934335.1	Streptococcus suis	PP422	RSDO_s	2075657
GCA_002961305.1	Streptococcus suis	1225	POLL_s	2527390
GCA_002960445.1	Streptococcus suis	2219	POJD_s	2202496
GCA_002096935.1	Streptococcus mitis	B_5756_13	NCVM_s	1896604
GCA_002096685.1	Streptococcus oralis subsp. dentisani	RH_13585_10	NCVA_s	1887959
GCA_002096655.1	Streptococcus oralis subsp. dentisani	RH_70047_11	NCUY_s	1955353
GCA_002096335.1	Streptococcus oralis subsp. dentisani	Y_5914_11	NCUW_s	1977550
GCA_002096595.1	Streptococcus oralis subsp. oralis	OD_311844-09	NCUR_s	1951174
GCA_002096535.1	Streptococcus oralis subsp. oralis	RH_1735_08	NCUN_s	2004683
GCA_002096445.1	Streptococcus oralis subsp. oralis	RH_57980_07	NCUK_s	1988397
GCA_002096435.1	Streptococcus oralis subsp. oralis	Y_11577_11	NCUI_s	1999772
GCA_002096365.1	Streptococcus oralis subsp. tigurinus	B_003802_10	NCUE_s	1933556
GCA_002096215.1	Streptococcus oralis subsp. tigurinus	OD_348934_12	NCUC_s	1852323
GCA_002014795.1	Streptococcus mitis	CCUG 63687	MUYO_s	2039754
GCA_001650315.1	Streptococcus sp. CCUG 49591	CCUG 49591	LVJM_s	1928655
GCA_900143575.1	Streptococcus suis	LS9N	LT671674_s	2561645
GCA_002093545.1	Streptococcus oralis subsp. tigurinus	AZ_8	LNVF_s	2126774
GCA_001182825.2	Streptococcus sp. X13SY08	X13SY08	LFYO_s	1548621
GCA_002005545.1	Streptococcus mitis	321A	LBMT_s	2110680
GCA_001814775.1	Streptococcus sp. HMSC067H01	HMSC067H01	KV817770_s	2052401
GCA_001810825.1	Streptococcus sp. HMSC076C08	HMSC076C08	KV802702_s	1998154
GCA_001579625.1	Streptococcus oralis	DD17	KQ970808_s	2148846
GCA_001579175.1	Streptococcus oralis	DD24	KQ970764_s	2129793
GCA_001579035.1	Streptococcus mitis	DD26	KQ970296_s	2122552
GCA_001579045.1	Streptococcus mitis	DD28	KQ970267_s	2169250
GCA_001579025.1	Streptococcus oralis	DD27	KQ970240_s	1956555
GCA_001578965.1	Streptococcus oralis	DD20	KQ969826_s	2086950
GCA_001578945.1	Streptococcus oralis	DD16	KQ969560_s	2052376
GCA_001578935.1	Streptococcus oralis	DD15	KQ969525_s	2001295
GCA_001578875.1	Streptococcus sp. DD13	DD13	KQ969510_s	1716726
GCA_001578885.1	Streptococcus sp. DD12	DD12	KQ969499_s	1676814
GCA_001578855.1	Streptococcus oralis	DD14	KQ969343_s	2031029

GCA_001578805.1	Streptococcus sp. DD10	DD10	KQ969171_s	2189983
GCA_001578795.1	Streptococcus gordonii	DD07	KQ969111_s	2232624
GCA_001578775.1	Streptococcus cristatus	DD08	KQ969067_s	2206539
GCA_001578705.1	Streptococcus oralis	DD05	KQ969042_s	1965030
GCA_000411475.1	Streptococcus sp. HPH0090	HPH0090	KE150464_s	1765113
GCA_000314775.2	Streptococcus sp. F0441	F0441	KB373321_s	2000650
GCA_000314795.2	Streptococcus sp. F0442	F0442	KB373315_s	2231248
GCA_000960105.1	Streptococcus mitis	UC5873	JYGU_s	1835325
GCA_000960085.1	Streptococcus mitis	UC921A	JYGT_s	1791991
GCA_000960025.1	Streptococcus mitis	SK137	JYGQ_s	1984405
GCA_000960005.1	Streptococcus mitis	OT25	JYGP_s	1915198
GCA_000959975.1	Streptococcus mitis	OP51	JYGO_s	1844175
GCA_000959945.1	Streptococcus mitis	COL85/1862	JYGM_s	1900154
GCA_000959885.1	Streptococcus cristatus	ATCC 49999	JYGJ_s	2026543
GCA_001069915.1	Streptococcus cristatus	1015_SOLI	JWGF_s	2019516
GCA_001069165.1	Streptococcus anginosus	1043_SSUI	JWEZ_s	1974848
GCA_001068775.1	Streptococcus pseudopneumoniae	1172_SPSE	JWAJ_s	2043463
GCA_001068835.1	Streptococcus pseudopneumoniae	1213_SPSE	JVYO_s	2024246
GCA_001068945.1	Streptococcus pseudopneumoniae	1271.rep1_SPSE	JVWC_s	2119135
GCA_001069865.1	Streptococcus pseudopneumoniae	144_SPSE	JVSM_s	1839663
GCA_001070715.1	Streptococcus pseudopneumoniae	163_SPSE	JVRR_s	2123243
GCA_001076615.1	Streptococcus pseudopneumoniae	294_SPSE	JVMO_s	2034729
GCA_001070815.1	Streptococcus pseudopneumoniae	315_SPSE	JVLX_s	1893353
GCA_001072315.1	Streptococcus pseudopneumoniae	330_SPSE	JVLI_s	2162940
GCA_001072375.1	Streptococcus pseudopneumoniae	342_SPSE	JVKV_s	1918053
GCA_001072925.1	Streptococcus pseudopneumoniae	434_SPSE	JVHE_s	2118581
GCA_001076775.1	Streptococcus pseudopneumoniae	445_SPSE	JVGV_s	2088167
GCA_001073085.1	Streptococcus pseudopneumoniae	469_SPSE	JVFV_s	2094752
GCA_001074155.1	Streptococcus pseudopneumoniae	74_SPSE	JUUZ_s	1772228
GCA_001074975.1	Streptococcus pseudopneumoniae	75_SPSE	JUUO_s	2094444
GCA_001074565.1	Streptococcus pseudopneumoniae	843_SPSE	JUQX_s	2085268
GCA_001074635.1	Streptococcus mitis	850_SMIT	JUQO_s	2084604
GCA_001074805.1	Streptococcus parasanguinis	886_SPAR	JUPI_s	2185482
GCA_001074825.1	Streptococcus pseudopneumoniae	888_SPSE	JUPG_s	2078715
GCA_001075875.1	Streptococcus oralis	900_SORA	JUOS_s	2098566
GCA_001075675.1	Streptococcus oralis	918_SORA	JUNW_s	1884524
GCA_000722755.1	Streptococcus mitis	SK578	JPFY_s	2080791
GCA_000722685.1	Streptococcus mitis	SK667	JPFV_s	2136987
GCA_000722695.1	Streptococcus mitis	SK629	JPFU_s	2213700
GCA_000722815.1	Streptococcus mitis	SK1126	JPFT_s	1932472
GCA_000235485.1	Streptococcus sp. oral taxon 058 str. F0407	F0407	JH378877_s	1848904

GCA_000212855.1	Streptococcus sanguinis SK355	SK355	GL890994_s	2370432
GCA_000212815.1	Streptococcus sanguinis SK49	SK49	GL890987_s	2279404
GCA_000195025.1	Streptococcus sanguinis SK330	SK330	GL878553_s	2415674
GCA_000187745.1	Streptococcus sp. M334	M334	GL732500_s	2207013
GCA_000187505.1	Streptococcus parasanguinis ATCC 903	ATCC 903	GL732452_s	2131530
GCA_000187445.1	Streptococcus sp. C150	C150	GL698454_s	2142483
GCA_000185265.1	Streptococcus oralis ATCC 49296	ATCC 49296	GL622184_s	2068336
GCA_000146585.1	Streptococcus mitis ATCC 6249	ATCC 6249	GL397180_s	1915891
GCA_000253155.1	Streptococcus oralis Uo5	Uo5	FR720602_s	1958690
GCA_900104285.1	Streptococcus sp. NLAE-zl-C503	NLAE-zl-C503	FNJT_s	1938063
GCA_000027165.1	Streptococcus mitis B6	B6	FN568063_s	2146611
GCA_900012395.1	Streptococcus suis	9401240	CZEF_s	2174179
GCA_001096185.1	Streptococcus pneumoniae	SMRU824	CRPU_s	2117177
GCA_001983955.1	Streptococcus oralis	S.MIT/ORALIS-351	CP019562_s	1966910
GCA_001683375.1	Streptococcus sp. oral taxon 064	W10853	CP016207_s	2013158
GCA_001560895.1	Streptococcus mitis	SVGS_061	CP014326_s	2167922
GCA_001553685.1	Streptococcus sp. oral taxon 431	F0610 (5-114)	CP014264_s	2177905
GCA_001281025.1	Streptococcus mitis	KCOM 1350 (= ChDC B183)	CP012646_s	1906344
GCA_000688775.2	Streptococcus sp. VT 162	VT 162	CP007628_s	2041142
GCA_000479315.1	Streptococcus sp. I-P16	I-P16	CP006776_s	2023580
GCA_000385925.1	Streptococcus oligofermentans AS 1.3089	AS 1.3089	CP004409_s	2142100
GCA_001113365.1	Streptococcus pneumoniae	SMRU2014	CKYA_s	2332265
GCA_001171885.1	Streptococcus pneumoniae	SMRU946	CKQD_s	2119625
GCA_001078705.1	Streptococcus sanguinis	2908	CDMW_s	2308610
GCA_000430305.1	Streptococcus mitis 17/34	17/34	ASZZ_s	1873245
GCA_000442175.1	Streptococcus tigurinus 2426	2426	ASXA_s	1882877
GCA_000385835.1	Streptococcus mitis 13/39	13/39	AQTU_s	2058881
GCA_002355895.1	Streptococcus sp. NPS 308	NPS 308	AP017652_s	1924728
GCA_000286295.1	Streptococcus salivarius K12	K12	ALIF_s	2426359
GCA_000279535.1	Streptococcus mitis SPAR10	SPAR10	ALCH_s	1758646
GCA_000259505.1	Streptococcus sp. SK643	SK643	AJMM_s	1860312
GCA_000223235.2	Streptococcus oralis SK313	SK313	AFUU_s	1888813
GCA_000223255.2	Streptococcus infantis SK970	SK970	AFUT_s	1945605
GCA_000223335.2	Streptococcus infantis X	X	AFUQ_s	1869505
GCA_000221165.2	Streptococcus mitis bv. 2 str. F0392	F0392	AFUO_s	1916998
GCA_000222725.2	Streptococcus parasanguinis SK236	SK236	AFUC_s	1988691
GCA_000222705.2	Streptococcus mitis bv. 2 str. SK95	SK95	AFUB_s	2020730
GCA_000220065.2	Streptococcus sp. oral taxon 056 str. F0418	F0418	AFQU_s	1943250
GCA_000215385.2	Streptococcus infantis SK1076	SK1076	AFNN_s	1744061
GCA_000215365.2	Streptococcus oralis SK255	SK255	AFNM_s	2005180

Table S2. References and truth for the synthetic metagenome samples.

Set	NCBI Accession	Taxon name	EzBioCloud name	Genome size	Truth
**1**	GCA_000164675.2	Streptococcus parasanguinis ATCC 15912	Streptococcus parasanguinis	2153652	15.00%
	GCA_000257765.1	Streptococcus anginosus subsp. whileyi CCUG 39159	Streptococcus anginosus subsp. whileyi	2294730	10.00%
	GCA_000187465.1	Streptococcus infantis ATCC 700779	Streptococcus infantis	1905984	5.00%
	GCA_000380005.1	Streptococcus didelphis DSM 15616	Streptococcus didelphis	1877438	5.00%
	GCA_000380025.1	Streptococcus entericus DSM 14446	Streptococcus entericus	2036468	15.00%
	GCA_000027165.1	Streptococcus mitis B6	FN568063_s	2146611	30.00%
	GCA_000185265.1	Streptococcus oralis ATCC 49296	GL622184_s	2068336	20.00%
**2**	GCA_000014485.1	Streptococcus thermophilus LMD-9	Streptococcus thermophilus	1864178	10.00%
	GCA_000007425.1	Streptococcus pyogenes MGAS315	Streptococcus pyogenes	1900521	15.00%
	GCA_000019025.1	Streptococcus pneumoniae Taiwan19F-14	Streptococcus pneumoniae	2112148	7.50%
	GCA_000007465.2	Streptococcus mutans UA159	Streptococcus mutans	2032925	7.50%
	GCA_000380045.1	Streptococcus marimammalium DSM 18627	Streptococcus marimammalium	1505444	10.00%
	GCA_000180055.1	Streptococcus downei F0415	Streptococcus downei	2239421	20.00%
	GCA_000463425.1	Streptococcus constellatus subsp. pharyngis C1050	Streptococcus constellatus subsp. pharyngis	1991156	5.00%
	GCA_000007265.1	Streptococcus agalactiae 2603V/R	Streptococcus agalactiae	2160267	5.00%
	GCA_000314795.2	Streptococcus sp. F0442	KB373315_s	2231248	20.00%
**3**	GCA_001578775.1	Streptococcus cristatus DD08	KQ969067_s	2206539	20.00%
	GCA_000722685.1	Streptococcus mitis SK667	JPFV_s	2136987	10.00%
	GCA_002005545.1	Streptococcus mitis 321A	LBMT_s	2110680	13.00%
	GCA_002096935.1	Streptococcus mitis B_5756_13	NCVM_s	1896604	5.00%
	GCA_001075675.1	Streptococcus oralis 918_SORA	JUNW_s	1884524	20.00%
	GCA_001579175.1	Streptococcus oralis DD24	KQ970764_s	2129793	13.00%
	GCA_002096595.1	Streptococcus oralis subsp. oralis OD_311844-09	NCUR_s	1951174	5.00%
	GCA_900012395.1	Streptococcus suis 9401240	CZEF_s	2174179	10.00%
	GCA_003934335.1	Streptococcus suis PP422	RSDO_s	2075657	4.00%
**4**	GCA_900095845.1	Streptococcus timonensis Marseille-P2915	Streptococcus timonensis	1925331	25.00%
	GCA_003595525.1	Streptococcus respiraculi HTS25	Streptococcus respiraculi	2067971	25.00%
	GCA_000423745.1	Streptococcus plurextorum DSM 22810	Streptococcus plurextorum	2103464	25.00%
	GCA_002953735.1	Streptococcus pluranimalium TH11417	Streptococcus pluranimalium	2065522	25.00%

Table S3. Species from the genus streptococcus covered in MetaPhlAn2.

Taxon Name	Domain	Phylum	Class	Order	Family	Genus
Streptococcus_agalactiae	Bacteria	p__Firmicutes	c__Bacilli	o__Lactobacillales	f__Streptococcaceae	g__Streptococcus
Streptococcus_anginosus	Bacteria	p__Firmicutes	c__Bacilli	o__Lactobacillales	f__Streptococcaceae	g__Streptococcus
Streptococcus_australis	Bacteria	p__Firmicutes	c__Bacilli	o__Lactobacillales	f__Streptococcaceae	g__Streptococcus
Streptococcus_caballi	Bacteria	p__Firmicutes	c__Bacilli	o__Lactobacillales	f__Streptococcaceae	g__Streptococcus
Streptococcus_canis	Bacteria	p__Firmicutes	c__Bacilli	o__Lactobacillales	f__Streptococcaceae	g__Streptococcus
Streptococcus_constellatus	Bacteria	p__Firmicutes	c__Bacilli	o__Lactobacillales	f__Streptococcaceae	g__Streptococcus
Streptococcus_criceti	Bacteria	p__Firmicutes	c__Bacilli	o__Lactobacillales	f__Streptococcaceae	g__Streptococcus
Streptococcus_cristatus	Bacteria	p__Firmicutes	c__Bacilli	o__Lactobacillales	f__Streptococcaceae	g__Streptococcus
Streptococcus_didelphis	Bacteria	p__Firmicutes	c__Bacilli	o__Lactobacillales	f__Streptococcaceae	g__Streptococcus
Streptococcus_downei	Bacteria	p__Firmicutes	c__Bacilli	o__Lactobacillales	f__Streptococcaceae	g__Streptococcus
Streptococcus_dysgalactiae	Bacteria	p__Firmicutes	c__Bacilli	o__Lactobacillales	f__Streptococcaceae	g__Streptococcus
Streptococcus_entericus	Bacteria	p__Firmicutes	c__Bacilli	o__Lactobacillales	f__Streptococcaceae	g__Streptococcus
Streptococcus_equi	Bacteria	p__Firmicutes	c__Bacilli	o__Lactobacillales	f__Streptococcaceae	g__Streptococcus
Streptococcus_ferus	Bacteria	p__Firmicutes	c__Bacilli	o__Lactobacillales	f__Streptococcaceae	g__Streptococcus
Streptococcus_gallolyticus	Bacteria	p__Firmicutes	c__Bacilli	o__Lactobacillales	f__Streptococcaceae	g__Streptococcus
Streptococcus_gordonii	Bacteria	p__Firmicutes	c__Bacilli	o__Lactobacillales	f__Streptococcaceae	g__Streptococcus
Streptococcus_henryi	Bacteria	p__Firmicutes	c__Bacilli	o__Lactobacillales	f__Streptococcaceae	g__Streptococcus
Streptococcus_ictaluri	Bacteria	p__Firmicutes	c__Bacilli	o__Lactobacillales	f__Streptococcaceae	g__Streptococcus
Streptococcus_infantarius	Bacteria	p__Firmicutes	c__Bacilli	o__Lactobacillales	f__Streptococcaceae	g__Streptococcus
Streptococcus_infantis	Bacteria	p__Firmicutes	c__Bacilli	o__Lactobacillales	f__Streptococcaceae	g__Streptococcus
Streptococcus_iniae	Bacteria	p__Firmicutes	c__Bacilli	o__Lactobacillales	f__Streptococcaceae	g__Streptococcus
Streptococcus_intermedius	Bacteria	p__Firmicutes	c__Bacilli	o__Lactobacillales	f__Streptococcaceae	g__Streptococcus
Streptococcus_lutetiensis	Bacteria	p__Firmicutes	c__Bacilli	o__Lactobacillales	f__Streptococcaceae	g__Streptococcus
Streptococcus_macacae	Bacteria	p__Firmicutes	c__Bacilli	o__Lactobacillales	f__Streptococcaceae	g__Streptococcus
Streptococcus_macedonicus	Bacteria	p__Firmicutes	c__Bacilli	o__Lactobacillales	f__Streptococcaceae	g__Streptococcus
Streptococcus_marimammalium	Bacteria	p__Firmicutes	c__Bacilli	o__Lactobacillales	f__Streptococcaceae	g__Streptococcus
Streptococcus_massiliensis	Bacteria	p__Firmicutes	c__Bacilli	o__Lactobacillales	f__Streptococcaceae	g__Streptococcus
Streptococcus_merionis	Bacteria	p__Firmicutes	c__Bacilli	o__Lactobacillales	f__Streptococcaceae	g__Streptococcus
Streptococcus_minor	Bacteria	p__Firmicutes	c__Bacilli	o__Lactobacillales	f__Streptococcaceae	g__Streptococcus
Streptococcus_mitis_oralis_pneumoniae	Bacteria	p__Firmicutes	c__Bacilli	o__Lactobacillales	f__Streptococcaceae	g__Streptococcus
Streptococcus_mutans	Bacteria	p__Firmicutes	c__Bacilli	o__Lactobacillales	f__Streptococcaceae	g__Streptococcus
Streptococcus_oligofermentans	Bacteria	p__Firmicutes	c__Bacilli	o__Lactobacillales	f__Streptococcaceae	g__Streptococcus
Streptococcus_orisratti	Bacteria	p__Firmicutes	c__Bacilli	o__Lactobacillales	f__Streptococcaceae	g__Streptococcus
Streptococcus_ovis	Bacteria	p__Firmicutes	c__Bacilli	o__Lactobacillales	f__Streptococcaceae	g__Streptococcus
Streptococcus_parasanguinis	Bacteria	p__Firmicutes	c__Bacilli	o__Lactobacillales	f__Streptococcaceae	g__Streptococcus
Streptococcus_parauberis	Bacteria	p__Firmicutes	c__Bacilli	o__Lactobacillales	f__Streptococcaceae	g__Streptococcus
Streptococcus_pasteurianus	Bacteria	p__Firmicutes	c__Bacilli	o__Lactobacillales	f__Streptococcaceae	g__Streptococcus

Streptococcus_peroris	Bacteria	p__Firmicutes	c__Bacilli	o__Lactobacillales	f__Streptococcaceae	g__Streptococcus
Streptococcus_porcinus	Bacteria	p__Firmicutes	c__Bacilli	o__Lactobacillales	f__Streptococcaceae	g__Streptococcus
Streptococcus_pseudopneumoniae	Bacteria	p__Firmicutes	c__Bacilli	o__Lactobacillales	f__Streptococcaceae	g__Streptococcus
Streptococcus_pseudoporcinus	Bacteria	p__Firmicutes	c__Bacilli	o__Lactobacillales	f__Streptococcaceae	g__Streptococcus
Streptococcus_pyogenes	Bacteria	p__Firmicutes	c__Bacilli	o__Lactobacillales	f__Streptococcaceae	g__Streptococcus
Streptococcus_ratti	Bacteria	p__Firmicutes	c__Bacilli	o__Lactobacillales	f__Streptococcaceae	g__Streptococcus
Streptococcus_salivarius	Bacteria	p__Firmicutes	c__Bacilli	o__Lactobacillales	f__Streptococcaceae	g__Streptococcus
Streptococcus_sanguinis	Bacteria	p__Firmicutes	c__Bacilli	o__Lactobacillales	f__Streptococcaceae	g__Streptococcus
Streptococcus_sobrinus	Bacteria	p__Firmicutes	c__Bacilli	o__Lactobacillales	f__Streptococcaceae	g__Streptococcus
Streptococcus_sp_2_1_36FAA	Bacteria	p__Firmicutes	c__Bacilli	o__Lactobacillales	f__Streptococcaceae	g__Streptococcus
Streptococcus_sp_AS14	Bacteria	p__Firmicutes	c__Bacilli	o__Lactobacillales	f__Streptococcaceae	g__Streptococcus
Streptococcus_sp_BS35b	Bacteria	p__Firmicutes	c__Bacilli	o__Lactobacillales	f__Streptococcaceae	g__Streptococcus
Streptococcus_sp_C150	Bacteria	p__Firmicutes	c__Bacilli	o__Lactobacillales	f__Streptococcaceae	g__Streptococcus
Streptococcus_sp_C300	Bacteria	p__Firmicutes	c__Bacilli	o__Lactobacillales	f__Streptococcaceae	g__Streptococcus
Streptococcus_sp_F0441	Bacteria	p__Firmicutes	c__Bacilli	o__Lactobacillales	f__Streptococcaceae	g__Streptococcus
Streptococcus_sp_F0442	Bacteria	p__Firmicutes	c__Bacilli	o__Lactobacillales	f__Streptococcaceae	g__Streptococcus
Streptococcus_sp_GMD4S	Bacteria	p__Firmicutes	c__Bacilli	o__Lactobacillales	f__Streptococcaceae	g__Streptococcus
Streptococcus_sp_GMD5S	Bacteria	p__Firmicutes	c__Bacilli	o__Lactobacillales	f__Streptococcaceae	g__Streptococcus
Streptococcus_sp_HPH0090	Bacteria	p__Firmicutes	c__Bacilli	o__Lactobacillales	f__Streptococcaceae	g__Streptococcus
Streptococcus_sp_I_G2	Bacteria	p__Firmicutes	c__Bacilli	o__Lactobacillales	f__Streptococcaceae	g__Streptococcus
Streptococcus_sp_I_P16	Bacteria	p__Firmicutes	c__Bacilli	o__Lactobacillales	f__Streptococcaceae	g__Streptococcus
Streptococcus_sp_M143	Bacteria	p__Firmicutes	c__Bacilli	o__Lactobacillales	f__Streptococcaceae	g__Streptococcus
Streptococcus_sp_M334	Bacteria	p__Firmicutes	c__Bacilli	o__Lactobacillales	f__Streptococcaceae	g__Streptococcus
Streptococcus_sp_oral_taxon_056	Bacteria	p__Firmicutes	c__Bacilli	o__Lactobacillales	f__Streptococcaceae	g__Streptococcus
Streptococcus_sp_oral_taxon_058	Bacteria	p__Firmicutes	c__Bacilli	o__Lactobacillales	f__Streptococcaceae	g__Streptococcus
Streptococcus_sp_oral_taxon_071	Bacteria	p__Firmicutes	c__Bacilli	o__Lactobacillales	f__Streptococcaceae	g__Streptococcus
Streptococcus_sp_SK140	Bacteria	p__Firmicutes	c__Bacilli	o__Lactobacillales	f__Streptococcaceae	g__Streptococcus
Streptococcus_sp_SK643	Bacteria	p__Firmicutes	c__Bacilli	o__Lactobacillales	f__Streptococcaceae	g__Streptococcus
Streptococcus_suis	Bacteria	p__Firmicutes	c__Bacilli	o__Lactobacillales	f__Streptococcaceae	g__Streptococcus
Streptococcus_thermophilus	Bacteria	p__Firmicutes	c__Bacilli	o__Lactobacillales	f__Streptococcaceae	g__Streptococcus
Streptococcus_thoraltensis	Bacteria	p__Firmicutes	c__Bacilli	o__Lactobacillales	f__Streptococcaceae	g__Streptococcus
Streptococcus_tigurinus	Bacteria	p__Firmicutes	c__Bacilli	o__Lactobacillales	f__Streptococcaceae	g__Streptococcus
Streptococcus_uberis	Bacteria	p__Firmicutes	c__Bacilli	o__Lactobacillales	f__Streptococcaceae	g__Streptococcus
Streptococcus_urinalis	Bacteria	p__Firmicutes	c__Bacilli	o__Lactobacillales	f__Streptococcaceae	g__Streptococcus
Streptococcus_vestibularis	Bacteria	p__Firmicutes	c__Bacilli	o__Lactobacillales	f__Streptococcaceae	g__Streptococcus
